# E3-14.7K Is Recruited to TNF-Receptor 1 and Blocks TNF Cytolysis Independent from Interaction with Optineurin

**DOI:** 10.1371/journal.pone.0038348

**Published:** 2012-06-04

**Authors:** Laura Klingseisen, Martin Ehrenschwender, Ulrike Heigl, Harald Wajant, Thomas Hehlgans, Stefan Schütze, Wulf Schneider-Brachert

**Affiliations:** 1 Institute for Medical Microbiology and Hygiene, University of Regensburg, Regensburg, Germany; 2 Division of Molecular Internal Medicine, Department of Internal Medicine II, University Hospital Würzburg, Würzburg, Germany; 3 Institute of Immunology, University of Regensburg, Regensburg, Germany; 4 Institute of Immunology, Christian-Albrechts-University of Kiel, Kiel, Germany; NIH-NCI, United States of America

## Abstract

Escape from the host immune system is essential for intracellular pathogens. The adenoviral protein E3-14.7K (14.7K) is known as a general inhibitor of tumor necrosis factor (TNF)-induced apoptosis. It efficiently blocks TNF-receptor 1 (TNFR1) internalization but the underlying molecular mechanism still remains elusive. Direct interaction of 14.7K and/or associated proteins with the TNFR1 complex has been discussed although to date not proven. In our study, we provide for the first time evidence for recruitment of 14.7K and the 14.7K interacting protein optineurin to TNFR1. Various functions have been implicated for optineurin such as regulation of receptor endocytosis, vesicle trafficking, regulation of the nuclear factor κB (NF-κB) pathway and antiviral signaling. We therefore hypothesized that binding of optineurin to 14.7K and recruitment of both proteins to the TNFR1 complex is essential for protection against TNF-induced cytotoxic effects. To precisely dissect the individual role of 14.7K and optineurin, we generated and characterized a 14.7K mutant that does not confer TNF-resistance but is still able to interact with optineurin. In H1299 and KB cells expressing 14.7K wild-type protein, neither decrease in cell viability nor cleavage of caspases was observed upon stimulation with TNF. In sharp contrast, cells expressing the non-protective mutant of 14.7K displayed reduced viability and cleavage of initiator and effector caspases upon TNF treatment, indicating ongoing apoptotic cell death. Knockdown of optineurin in 14.7K expressing cells did not alter the protective effect as measured by cell viability and caspase activation. Taken together, we conclude that optineurin despite its substantial role in vesicular trafficking, endocytosis of cell surface receptors and recruitment to the TNFR1 complex is dispensable for the 14.7K-mediated protection against TNF-induced apoptosis.

## Introduction

Tumor necrosis factor (TNF) is a highly pleiotropic cytokine with critical functions in diverse cellular events ranging from proliferation to inflammation and induction of apoptosis [1]. It is furthermore of outstanding importance for the eradication of intracellular pathogens. *Vice versa*, intracellular pathogens obviously require sophisticated strategies to escape from TNF-mediated cytotoxic effects and the host immune system. Several proteins encoded in the early region 3 (E3) of adenoviruses facilitate infection or promote persistence in hosts by regulating the activity of cytokines [2]. Especially E3-14.7K (14.7K) efficiently prevents TNF-mediated cytotoxicity by blocking TNF-receptor 1 (TNFR1) internalization [3], [4].

Under physiological circumstances, TNFR1 is internalized upon ligand binding via clathrin-coated vesicles within minutes [5]. In earlier studies, we provided evidence that TNFR1 internalization not only functions as a “switch-off” for signaling events by receptor degradation and/or recycling [6]. In fact, TNFR1 internalization constitutes a *conditio sine qua non* for assembly of the death inducing signaling complex (DISC), a crucial event followed by caspase activation and induction of apoptotic cell death. Interestingly, activation of the proinflammatory and antiapoptotic nuclear factor κB (NF-κB) pathway is independent from TNFR1 internalization [4]. To date, the molecular mechanism of 14.7K mediated TNF-resistance is poorly understood, especially as several attempts to demonstrate interaction of 14.7K with TNFR1 complex failed [4], [7].

Potentially, intracellular binding proteins provide an indirect linkage between 14.7K and TNFR1. In a previous study, four 14.7K interacting proteins were identified in a yeast-two-hybrid screen, including inhibitor of kappa B kinase γ (IKKγ), the small GTPase RagA, apoptosis inducing factor (AIF) and optineurin (OPTN) [8], [9]. Beside the role of the latter in wide-angle glaucoma [10], optineurin is recruited to the TNFR1 complex, where it negatively regulates the NF-κB pathway [11], alters intracellular traffic of vesicels [12] and impairs endocytosis of cell surface receptors [13]. Regarding the properties of this molecule, optineurin potentially represents the molecular link between 14.7K and the TNFR1 complex. This led us to the hypothesis that protection against TNF-mediated cytotoxic effects requires recruitment of both optineurin and 14.7K to TNFR1 complex.

To analyze the functional significance of 14.7K interaction with optineurin we took advantage of a previously characterized 14.7K mutant. A C-terminal point mutation (C119S, designated 14.7K PM) affects structural integrity and abolishes protection against TNF-mediated cytotoxic effects [4], [14].

We demonstrate in this study that 14.7K PM not only exhibits loss of protection against TNF-mediated cytotoxic effects, but is also defective in optineurin binding. Whether susceptibility is due to functional defects of 14.7K PM caused by the point mutation or absence of optineurin remained elusive and required generation of 14.7K mutants with intact optineurin binding. Our results suggest that 14.7K-mediated TNF-resistance is not associated with absence or presence of optineurin, but critically depends on expression of 14.7K wilde-type protein. Taken together, we conclude that optineurin binds 14.7K as part of the TNFR1 complex, but is despite its role in vesicular trafficking and endocytosis of cell surface receptors dispensable for the 14.7K-mediated protection against TNF-induced cytotoxicity.

## Results

The protective effect of 14.7K against TNF-mediated cytotoxicity has been demonstrated in several studies [7], [14], [15] and was attributed to impaired TNFR1 internalization and subsequent absence of death inducing signaling complex (DISC) formation [4]. However, the molecular mechanism underlying this phenomenon is to date still unclear. Earlier studies suggested a crucial role for cellular 14.7K interacting proteins [8], [9]. Among previously identified candidate proteins, optineurin was reported to modulate the anti-apoptotic effect of 14.7K [9], [16]. As a component of the TNFR1 complex, optineurin is furthermore involved in regulation of NF-κB signaling by competing with IKKγ for ubiquitinated RIP1 [11], [17]. In addition, a role for optineurin in endo- and exocytotic vesicle trafficking has been demonstrated [12], [18].

In this study, we aimed to understand the physiological relevance of 14.7K-optineurin interaction in 14.7K-mediated TNF-resistance. As optineurin is part of the TNFR1 complex, we tested the hypothesis that both optineurin and 14.7K are upon ligand binding recruited to TNFR1 and are essential for 14.7K-mediated TNF-resistance.

### 14.7K is Part of TNFR1 Complex and Mediates Protection Against TNF-induced Cytotoxicity

**Figure 1:**
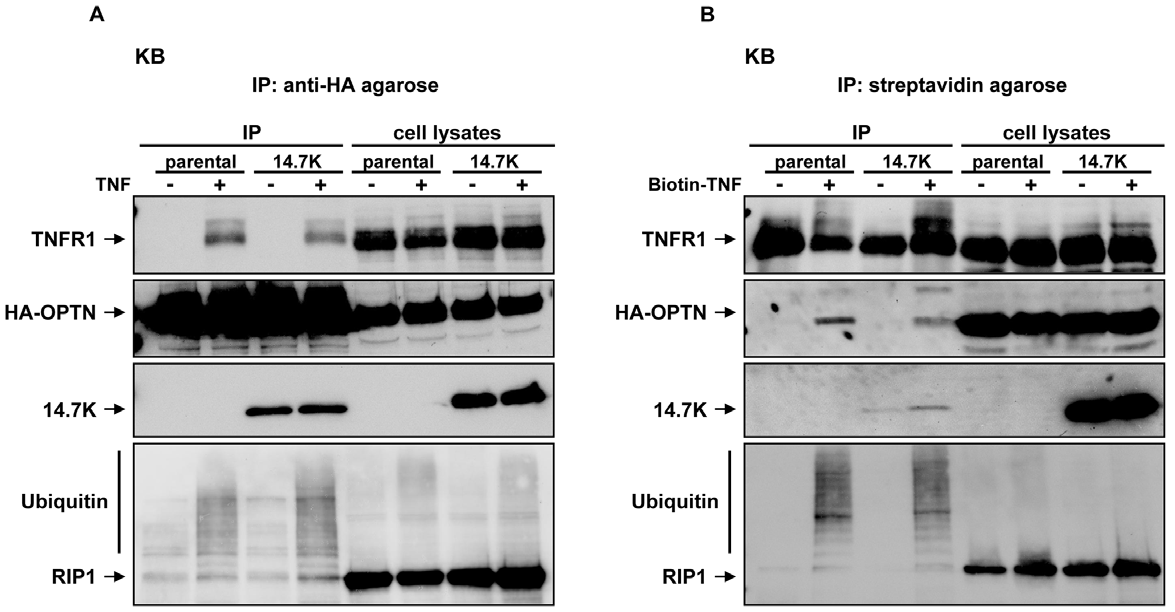
Optineurin and 14.7K are recruited to TNFR1 complex. Parental KB cells and KB cells stably expressing 14.7K were transfected with 30 µg HA-tagged optineurin. 24 hours after transfection, cells were challenged with 50 ng/mL TNF (A) or biotinylated TNF (B) for 10 min or left untreated. Proteins associated with HA-tagged optineurin (A) or biotinylated TNF (B) were analyzed together with the corresponding lysates for the presence of TNFR1, optineurin, 14.7K and RIP1.

Immunoprecipitation of 14.7K-optineurin complexes were performed in KB cells and stably transduced 14.7K expressing variants thereof (designated KB/14.7K). Before immunoprecipitation, cells were transfected with HA-tagged optineurin and 24 hours after transfection treated with 50 ng/mL TNF (Figure 1A) or biotinylated TNF (Figure 1B) for 10 min or left untreated. Cell lysates were subjected to immunoprecipitation with anti-HA agarose or streptavidin-coupled agarose. To control specificity when precipitating the ligand, 50 ng/mL biotin-TNF was added in excess to cell lysates from unstimulated cells.

In Western blot analysis we showed that 14.7K, TNFR1 and RIP1 coimmunoprecipitated with HA-tagged optineurin, indicating formation of a protein complex (Figure 1A). This result was in good accordance with previous studies, describing optineurin as a 14.7K and RIP1 interacting protein [8], [11]. Of note, formation of the 14.7K-optineurin complex was also detectable without stimulation while recruitment to the TNFR1 complex and ubiquitination of TNFR1-associated RIP1 were dependent on TNF treatment. Interestingly, even though the predicted binding sites of 14.7K and RIP1 were both located in the C-terminus of optineurin, 14.7K obviously did not disturb interaction of optineurin and RIP1 [10].

To further substantiate our findings and to exclude formation of two independent protein complexes (cytosolic 14.7K-optineurin complex on the one hand and a optineurin-RIP1-TNFR1 complex on the other hand) we also performed reverse immunoprecipitations with biotin-labeled TNF and streptavidin-coupled agarose (Figure 1B). Analogous to our previous experiments, we precipitated 14.7K, RIP1 and TNFR1 together with biotinylated TNF. In sum, these results indicate formation of a ligand-inducible complex at TNFR1 containing optineurin, 14.7K and RIP1. Interestingly, we also detected a faint 14.7K-band in immunoprecipitations of untreated cells (Figure 1B, lane 3), raising the possibility of a TNFR1-14.7K complex even in the absence of TNF-induced recruitment of optineurin to TNFR1. Technically, precipitation of this complex was feasible because 50 ng/mL of biotinylated TNF were added to lysates of unstimulated cells as specificity control. Therefore, optineurin-mediated recruitment is potentially not an exclusive mechanism of 14.7K-association with TNFR1 complex. It may rather constitute an additional route for 14.7K to TNFR1, possibly with the objective to modulate signaling pathways with optineurin involvement.

### Non-protective 14.7K PM Exhibits Loss of Interaction with Optineurin

**Figure 2:**
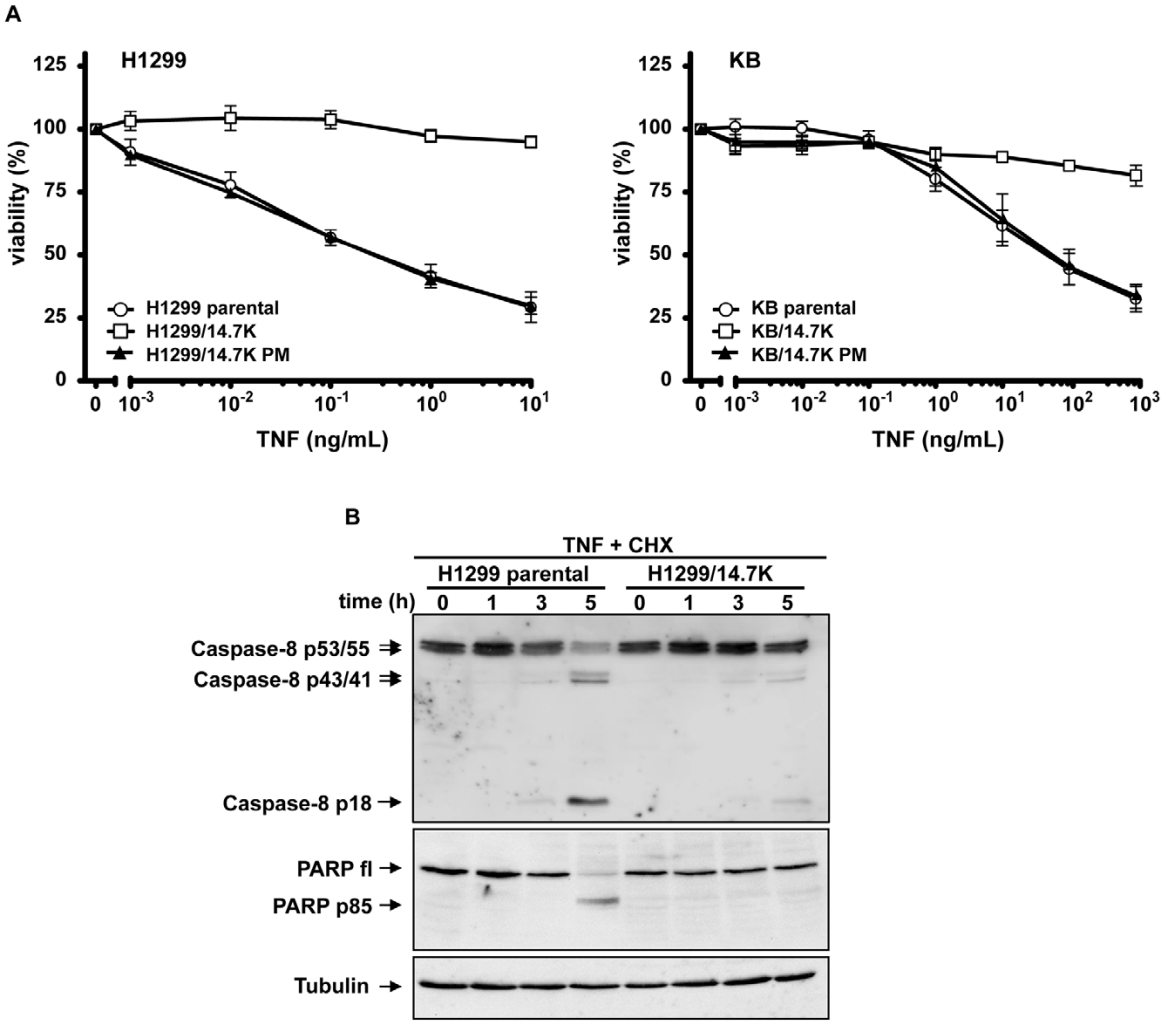
14.7K protects H1299 and KB cells from TNF-induced apoptosis. (A) H1299 and KB cells expressing either wild-type 14.7K (open squares) or 14.7K PM (black triangles) or untransfected cells (open circles) were seeded in triplicates in 96-well plates. The next day, cells were sensitized with cycloheximide (H1299 cells 12.5 µg/mL, KB cells 2.5 µg/mL) and treated with increasing amounts of TNF (H1299 cells 0.001–10 ng/mL, KB cells 0.001–1000 ng/mL). Dead cells were removed by washing with PBS followed by staining of viable cells with crystal violet. (B) Caspase activation was analyzed in CHX-sensitized (12.5 µg/mL for 1 hour) H1299 parental and H1299/14.7K cells. After stimulation with TNF for the indicated time, cells were harvested, lysed and subjected to Western blotting.

Several studies characterized 14.7K as a general inhibitor of TNF-induced cytolysis [7], [14], [15]. Structural integrity of the protein seems essential as the C-terminal point mutation C119S, referred to as 14.7K PM, resulted in loss of function [14]. In good accordance with previous data, H1299 and KB cells expressing wild-type 14.7K were resistant to TNF-mediated cytotoxicity even in the presence of cycloheximide (CHX) as sensitizing agent, displaying only about 20% apoptotic cells when challenged with TNF in doses far exceeding physiological levels (Figure 2A). In sharp contrast, 14.7K PM expressing cells of both cell lines were as susceptible as untransduced parental cell lines and displayed about 80% cytolysis. These findings were also reflected by detectable caspase-8 activation in H1299 parental cells and subsequent poly(ADP-ribose)-polymerase 1 (PARP) cleavage (Figure 2B). Both events were absent in H1299/14.7K cells.

**Figure 3:**
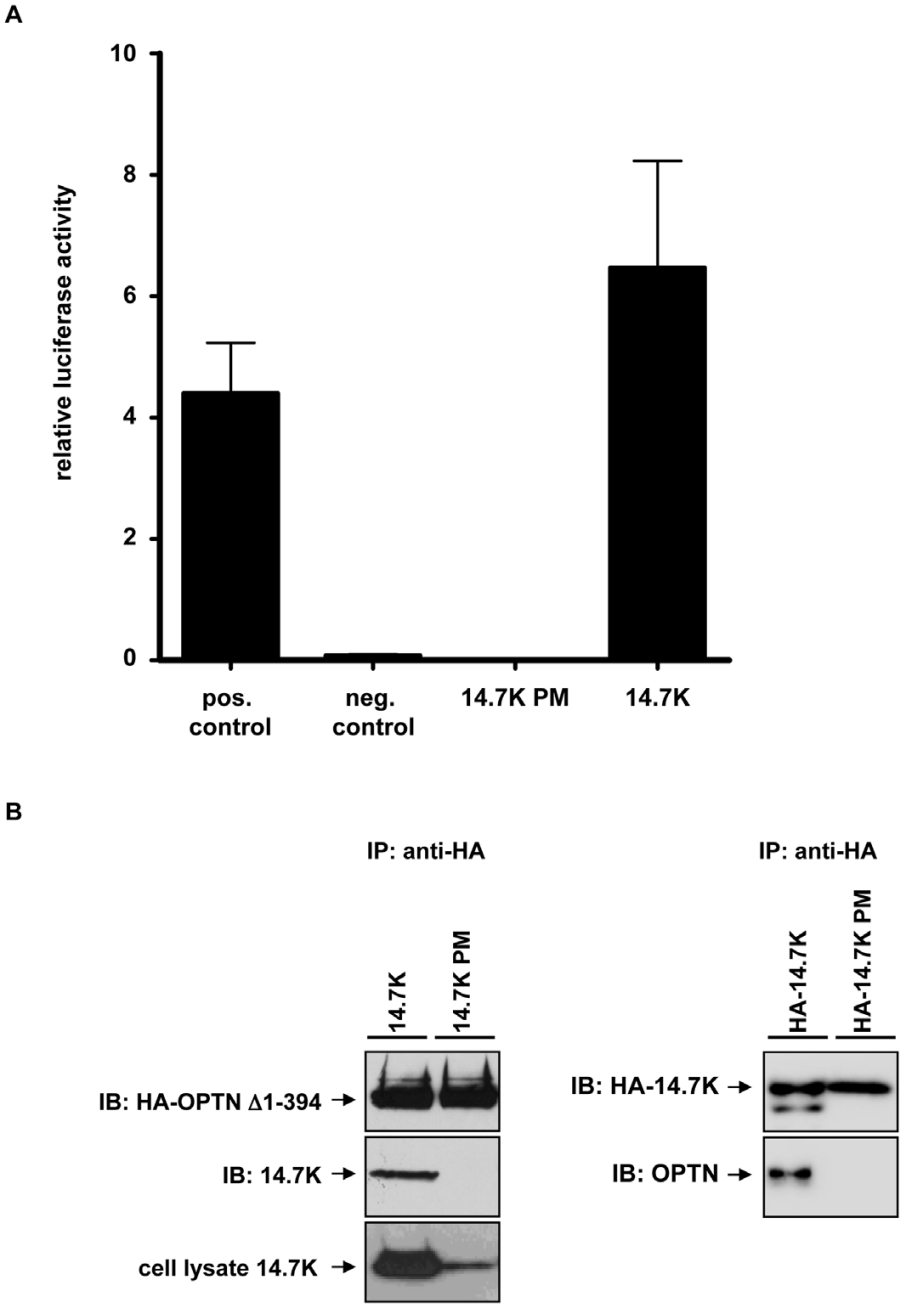
14.7K PM fails to interact with optineurin. (A) HEK293 cells were transfected with 200 ng of either wild-type 14.7K or 14.7K PM cloned in pAD expression plasmid and OPTN Δ1-394 along with the reporter luciferase firefly (pFR-luc) and a transfection control luciferase renilla (pRL-TK). 48h after transfection, luciferase activity in cell lysates was measured. The relative luciferase activities are expressed as ratio of the reporter luciferase to the transfection control (n = 3). (B) For immunoprecipitation experiments, HEK293 cells were transfected with 5 µg of either wild-type 14.7K or 14.7K PM along with OPTN Δ1-394 cloned in the expression plasmid pQCXIP (left panel). 48 hours after transfection cell lysates were subjected to immunoprecipitation using anti-HA agarose. Immunoprecipitates were analyzed by Western blotting using HA and 14.7K antibodies. Detection of 14.7K in corresponding lysates required immunoprecipitation with 14.7K antibody due to low expression level of 14.7K PM. To achieve comparable levels of 14.7K and 14.7K PM (right panel), HEK293 cells were transfected with 5 µg HA-tagged wildtype 14.7K or 15 µg of HA-tagged 14.7K PM along with 5 µg optineurin.

In order to examine a possible correlation between optineurin-14.7K interaction and TNF-resistance, we analyzed in a first approach the binding of 14.7K and 14.7K PM to optineurin in a mammalian-two-hybrid assay, using an optineurin construct (OPTN Δ1-394) with the identified 14.7K binding site [8] as bait (Figure 3A). As expected, 14.7K interacted with OPTN Δ1-394 as measured by reporter luciferase activity. However, the non-protective 14.7K PM (see also Figure 2) failed to induce luciferase activity, a clear indicator for loss of optineurin binding.

These predicted interactions were confirmed in coimmunoprecipitation experiments (Figure 3B). Cell lysates from HEK293 cells transfected with HA-tagged optineurin and 14.7K or 14.7K PM were subjected to immunoprecipitation with anti-HA agarose. 14.7K was detectable after precipitation of HA-tagged optineurin, thereby confirming interaction of the two proteins. However, due to low expression levels of 14.7K PM, assessment of total 14.7K or 14.7K PM expression was only achieved by immunoprecipitation of these proteins from the cell lysate. Consequently, to exclude the possibility that weak expression of 14.7K PM may be causative for the observed loss of optineurin interaction, we also performed a reverse coimmunoprecipitation in HEK293 cells transfected with HA-tagged 14.7K and wildtype optineurin (Figure 3B). In this approach, we achieved similar precipitation levels of 14.7K and 14.7K PM. Cell lysates were subjected to immunoprecipitation with anti-HA-agarose and precipitates immunoblotted for HA-14.7K and optineurin. Both experimental setups verified the non-protective 14.7K PM to be defective in optineurin-binding, which was in good accordance with our previous results. At this point, our data suggested a fundamental role for the TNFR1-associated 14.7K-optineurin complex in mediating TNF-resistance.

### Generation and Characterization of 14.7K Mutants

**Figure 4:**
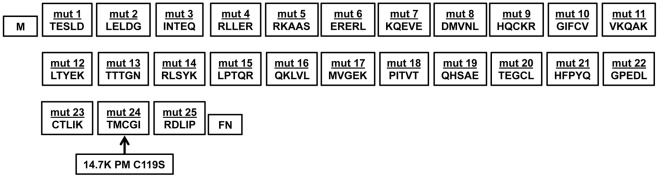
Generation of sequential mutants of 14.7K. The top row gives the names of the generated mutants. The bottom row displays the amino acid sequence of 14.7K replaced by a five amino acid Flag-tag (DYKDE). The arrow points to the C119S substitution, which was designated as 14.7K PM.

**Figure 5:**
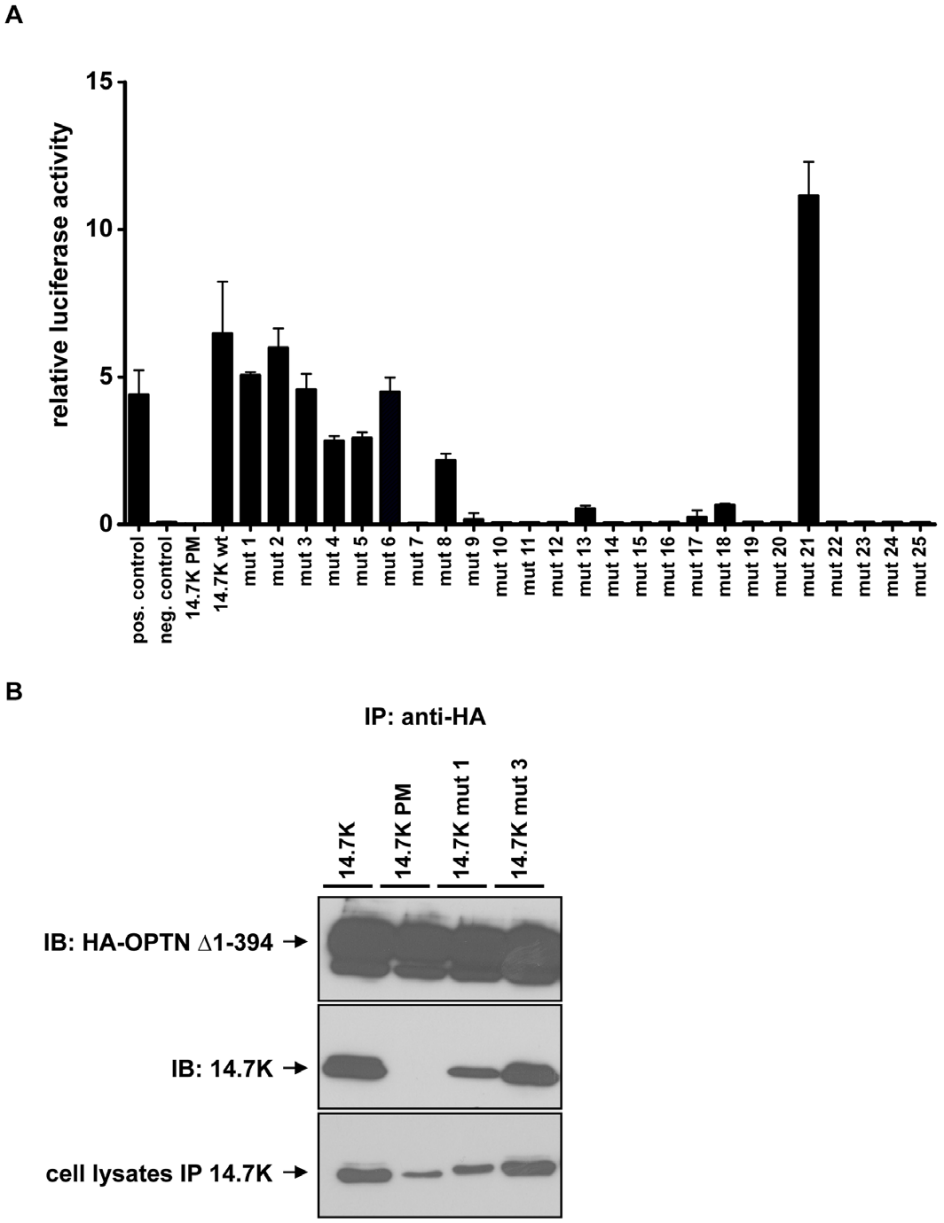
Identification of 14.7K-mutants interacting with optineurin. (A) HEK293 cells were transfected with mutants of 14.7K cloned in pCMV-AD and OPTN Δ1-394 (pCMV-BD) along with the reporter luciferase firefly (pFR-luc) and a transfection control luciferase renilla (pRL-TK). 48 h after transfection cell lysates were prepared for luciferase measurement. The relative luciferase activities are indicated as ratio of reporter luciferase to transfection control (n = 3). (B) HEK293 cells were transfected with either 5 µg 14.7K wild-type or indicated derivates along with 5 µg HA-tagged OPTN Δ1-394 and subjected to immunoprecipitation using HA-agarose. Immunoprecipitates were analyzed for optineurin-associated proteins by Western blotting. Detection of 14.7K PM from the corresponding lysates required immunoprecipitation to overcome low expression levels.

The failure of 14.7K PM to protect against TNF could be attributed to mutation-related loss of function of 14.7K PM or absence of its interaction partner optineurin. In consequence, individual characterization of the contribution of optineurin and 14.7K to TNF-resistance required generation of suitable 14.7K mutants, ideally with no intrinsic protection against TNF but conserved optineurin binding. Accordingly, we performed a systematic mutagenesis and sequentially replaced five amino acids by a Flag-tag sequence (DYKDE), covering the entire 14.7K gene (Figure 4). All generated 14.7K mutants were characterized regarding their capability to bind optineurin using a mammalian-two-hybrid screen (Figure 5A). With one exception, mutations in the C-terminus resulted in loss of optineurin binding, thereby confirming earlier studies stating that the C-terminal region was essential for protein-protein interactions [14], [19]. Consequently, to maximize the probability to obtain a structural integer 14.7K mutant without TNF-resistance, we choose two N-terminal mutants, designated as “14.7K mut 1” and “14.7K mut 3”. Optineurin-14.7K interaction of the selected mutants was additionally validated in coimmunoprecipitation experiments (Figure 5B).

### Optineurin Binding to 14.7K is not Sufficient to Mediate TNF-resistance

**Figure 6:**
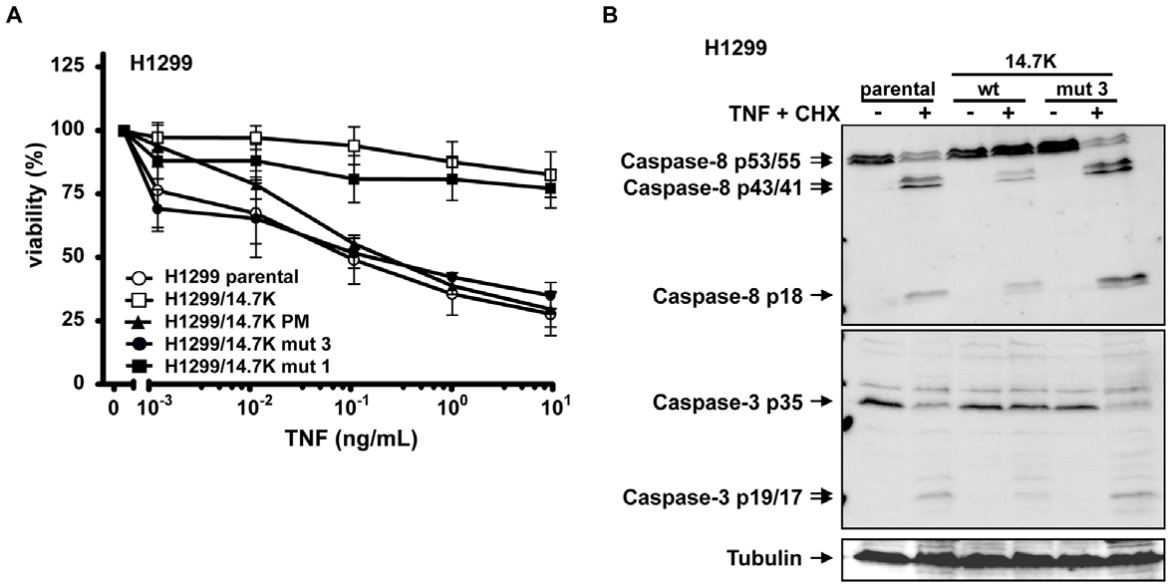
Optineurin binding to 14.7K is not sufficient for protection against TNF. (A) H1299 cells expressing indicated 14.7K variants were seeded in triplicates in 96-well plates and treated with 12.5 µg/mL CHX and increasing amounts of TNF (0.001–10 ng/mL) the next day. Dead cells were removed by washing with PBS followed by staining of viable cells with crystal violet. (B) H1299 cells expressing either 14.7K, 14.7K mut 3 or untransfected cells were treated (right panel) or not (left panel) with CHX (12.5 µg/mL) followed by stimulation with 20 ng/mL TNF for 5 h. Lysates were analyzed by Western blotting with caspase-8 and caspase-3 antibodies. Tubulin was used as loading control.

Selected 14.7K mutants with retained optineurin binding capacity were stably expressed in H1299 cells and assessed for TNF-protection (Figure 6A). 14.7K mut 1 exhibited a comparable degree of protection as 14.7K wild-type protein. This made the construct unfeasible for tracing back TNF-resistance to either 14.7K or 14.7K-optineurin complex and it was therefore excluded. However, 14.7K mut 3 failed to rescue cells after challenge with TNF although optineurin binding was retained, making it a suitable candidate for further investigations.

Again, results from cytotoxicity assays were concordant with results of caspase activation analysis. Following TNF-stimulation, H1299 and H1299/14.7K mut 3 cells showed cleavage of initiator and effector caspases as correlate for ongoing apoptotic cell death (Figure 6B), which was not detectable in H1299/14.7K cells. These findings were consistent with the observations from cytotoxicity assays shown in Figure 2A and with previous studies, demonstrating a blockade of caspase-8 cleavage in 14.7K expressing cells [4]. Together, these results indicated that interaction of 14.7K with optineurin is surprisingly not involved in inhibition of caspase-8 activation.

### Optineurin Knockdown does not Affect 14.7K-mediated Protection Against TNF

So far, our results suggested that optineurin is dispensable for TNF-resistance mediated by 14.7K. However, it had to be excluded that lost protection of 14.7K mut 3 is due to the N-terminal exchange of five amino acids and resulting impaired structural integrity, in case of the 14.7K protein a commonly encountered phenomenon [14].

**Figure 7:**
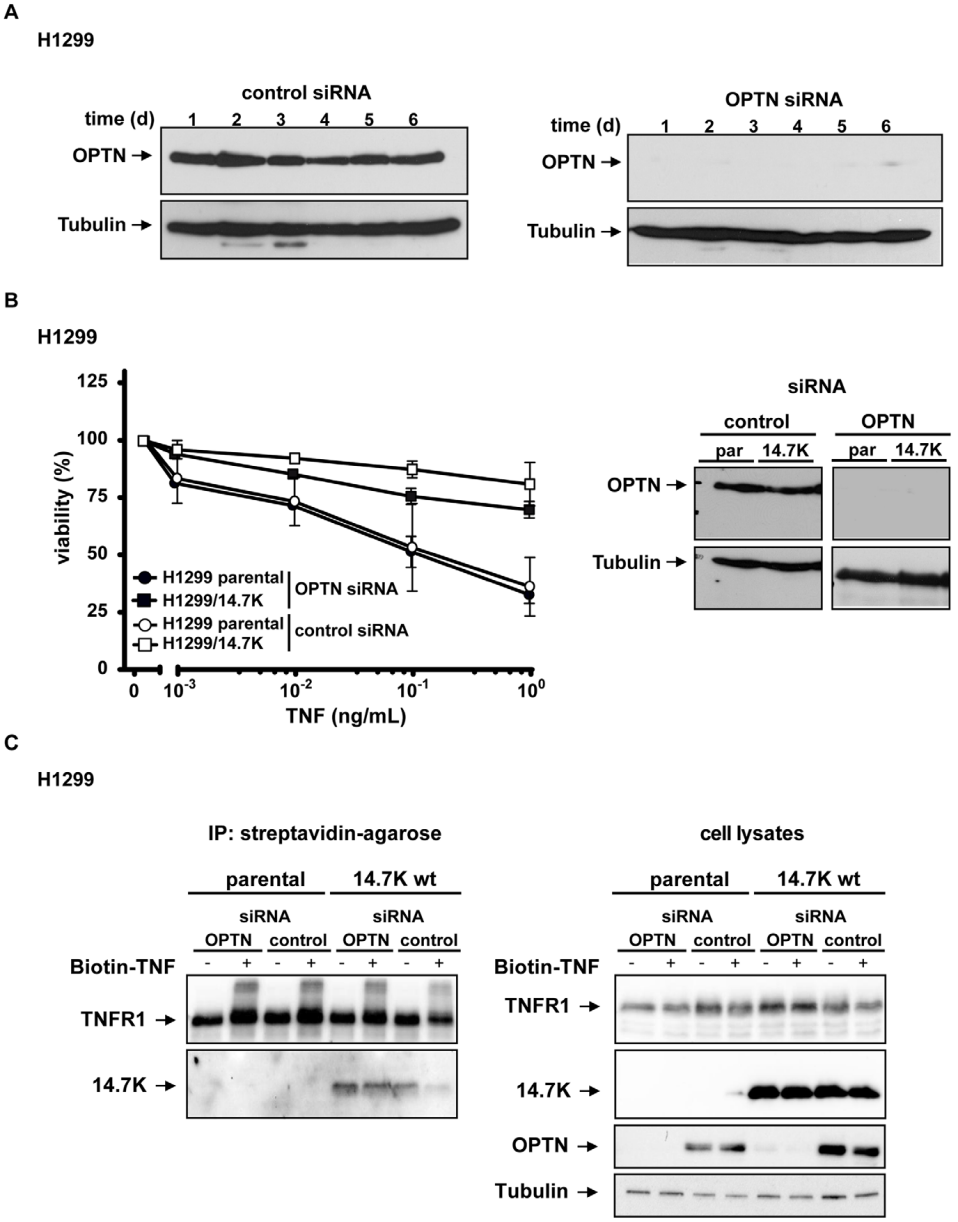
14.7K-mediated protection against TNF cytotoxicity and recruitment to TNFR1 is independent from endogenous optineurin. (A) Knockdown efficacy of optineurin-specific siRNA was confirmed by Western blotting and lasted at least for 6 days (B) H1299 parental or 14.7K expressing cells transfected with 100 pmol optineurin specific or control siRNA were seeded in 96-well plates and after 48 h challenged with increasing amounts of TNF (0.001–1 ng/mL) in the presence of CHX (12.5 µg/mL). Cell viability was assayed by staining with crystal violet. Knockdown of endogenous protein was confirmed by Western blotting. (C) Cells were seeded in a 10 cm culture plate, transfected with 600 pmol optineurin-specific siRNA or control siRNA and treated with 50 ng/mL biotin-labeled TNF 24 hours after transfection. Proteins associated with biotinylated TNF were analyzed for the presence of TNFR1 and 14.7K. Cell lysates confirmed efficient knockdown of optineurin in the corresponding samples.

We therefore used siRNA-mediated knockdown of optineurin to investigate the impact on 14.7K-mediated TNF-resistance. Efficacy and duration of siRNA-mediated optineurin knockdown was measured and lasted at least over a period of 6 days (Figure 7A). H1299 and H1299/14.7K cells treated with optineurin-specific or control siRNA were challenged in a cytotoxicity assay with increasing amounts of TNF in the presence of the sensitizing agent CHX (Figure 7B). Knockdown efficacy of siRNA transfection was ensured by Western blotting. H1299 cells expressing 14.7K were protected against TNF-mediated cytotoxicity in the presence and absence of optineurin, excluding an essential contribution of optineurin to the protective effect. Although knockdown of optineurin led to a slight, statistically not significant (p>0.05, Kruskal-Wallis test) loss of cell viability upon TNF treatment (Figure 7B, approx. 9% in H1299 parental cells and 13% in H1299/14.7K cells), wildtype 14.7K expressing cells were clearly protected against cytotoxic effects of TNF in the absence of optineurin. As TNF cytolysis was evident in H1299 parental cells in absence of optineurin, it was likely that this molecule is not involved in TNFR1-associated apoptotic mechanisms either.

As we noticed presumably optineurin-independent formation of TNFR1-14.7K complexes in our previous immunoprecipitation experiments (Figure 1B), we next addressed the role of optineurin as a crucial adaptor to convey 14.7K to TNFR1 complex. Immunoprecipitation experiments in KB cells treated with optineurin-specific siRNA and stimulated with biotin-labeled TNF (Figure 7C) demonstrated 14.7K-recruitment to TNFR1 complex independent of optineurin expression and TNF-stimulation. The faint protein band of 14.7K in the last lane of the immunoprecipitation group is likely to be due to lower precipitation of TNFR1 in that sample. Knockdown efficacy of optineurin was again assured by Western blotting.

Together, these results strengthened our hypothesis of TNF- and optineurin independent TNFR1-14.7K protein complex formation.

**Figure 8:**
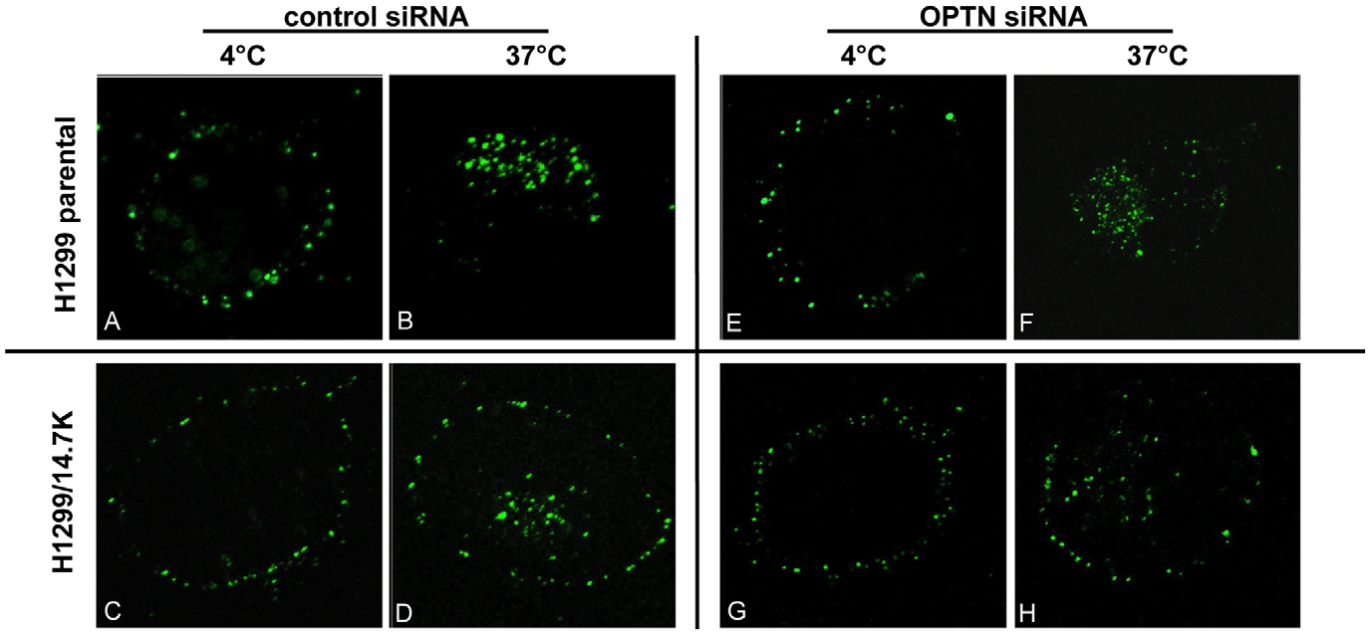
Blockade of 14.7K-mediated TNFR1 internalization persists in optineurin-knockdown cells. Confocal microscopy of H1299 parental or H1299 14.7K expressing cells 48 h after transfection with 100 pmol optineurin-specific or control siRNA. Cells were labeled with biotin-TNF/strepavidin-FITC complexes for one hour on ice and analyzed immediately for TNFR1 internalization (pictures A, C, E, G) or shifted to 37°C for another hour before microscopy (pictures B, D, F, H). Pictures were acquired using a Zeiss LSM 510, magnification 630-fold. A representative experiment of three independent experiments is shown.

The TNF-resistant phenotype of optineurin knockdown cells was also confirmed by the persistent blockade of TNFR1 internalization. Confocal microscopy of H1299 cells stably transfected with 14.7K wild-type protein exhibited blocked TNFR1 internalization, irrespective of the absence or presence of optineurin (Figure 8). Taken together, a significant contribution of optineurin to the 14.7K-mediated protective effect was unlikely.

### Optineurin is not Required for TNFR1-associated NF-κB Activation

**Figure 9:**
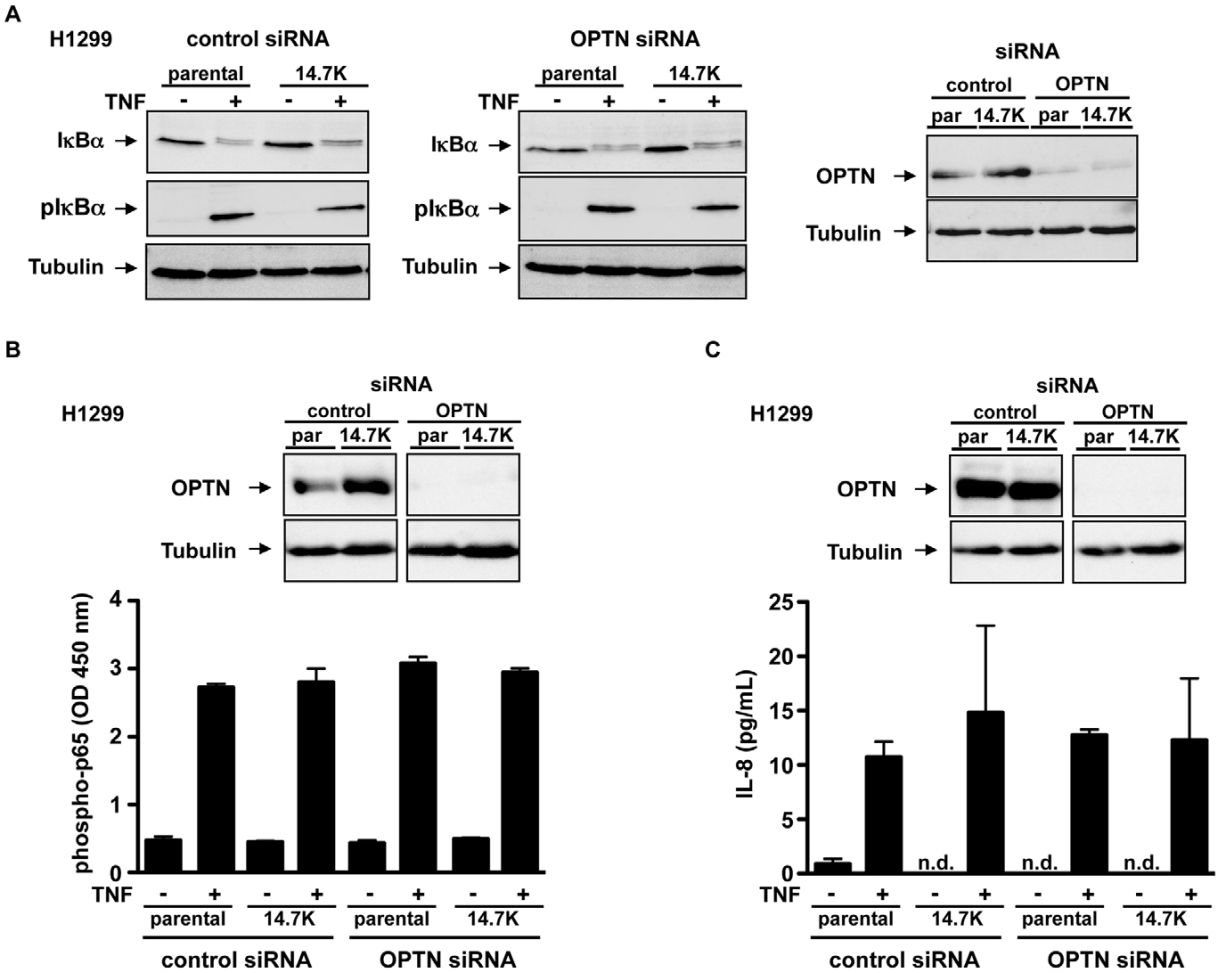
14.7K and optineurin do not affect NF-kB activation. H1299 parental or 14.7K expressing cells were transfected with 100 pmol optineurin-specific siRNA or control siRNA. Knockdown of optineurin was confirmed by Western blotting. (A) Cells were challenged with TNF (20 ng/mL) for 5 min. Cell lysates were subjected to Western blot analysis using antibodies specific for IκBα, phospho-IκBα and tubulin (loading control). (B) Cells were challenged with TNF (20 ng/mL) for 10 minutes. Cell lysates were subjected to phospho-p65 ELISA and analyzed at OD 450 nm. (C) Cells were challenged with TNF (20 ng/mL) for 24 hours. Supernatants were subjected to IL-8 ELISA. n.d.  =  not detectable.

As ligand binding to TNFR1 under physiological conditions primarily induces proinflammatory NF-κB signaling, we consequently examined the effects of optineurin knockdown on activation of NF-κB inhibitor IκBα. Phosphorylation of IκBα acts as a trigger for its degradation, leading to nuclear translocation of p65/p50 heterodimers [20]. To determine the effect of optineurin on IκBα phosphorylation in 14.7K expressing cells, H1299 and H1299/14.7K cells were transfected with optineurin-specific or control siRNA. 48 hours after transfection cells were treated with TNF for 5 min and cell lysates were analyzed by Western blotting with antibodies specific for IκBα and phospho-IκBα (Figure 9A). Knockdown efficacy was again confirmed by Western blotting. Stimulation of cells with TNF resulted in rapid phosphorylation of IκBα in all cell lines, irrespective of siRNA transfection. These findings were complemented by measurement of the activated NF-κB subunit phospho-p65 and the NF-κB-controlled target gene IL-8. Phosphorylation and degradation of IκBα leads to the release and phosphorylation of NF-κB subunit p65 which in return translocates into the nucleus to initiate the production of pro-inflammatory cytokines such as IL-8 [20]. In good agreement with unaltered IκBα phosphorylation, neither phosphorylation of NF-κB subunit p65 (Figure 9B) nor production of IL-8 (Figure 9C) was affected by knockdown of optineurin. As reported earlier, the activation of NF-κB pathway was not affected by presence or absence of 14.7K [4]. Together, these observations suggest that neither absence of optineurin nor presence of 14.7K did alter NF-κB activation. In the light of our findings, optineurin was neither essential for the antiapoptotic effect of 14.7K nor for the activation of NF-κB pathway in 14.7K expressing cells.

## Discussion

Immune escape mechanisms are of vital importance for intracellular pathogens. In this study, we report association of optineurin and 14.7K with TNFR1 complex. The protective effect of 14.7K against TNF-mediated cytolysis is solely dependent on presence of functional 14.7K, not on interaction with its binding partner optineurin. Furthermore, recruitment of 14.7K to TNFR1 does not affect TNF-induced proinflammatory NF-κB signaling.

The phenomenon of 14.7K-mediated protection against TNF was investigated in an earlier study of our group. Essentially, 14.7K prevents TNFR1 internalization and thereby abolishes subsequent formation of the death inducing signaling complex, whereas proinflammatory TNFR1 signaling involving RIP1 and TRAF2 was not affected [4]. At that time we failed to identify the molecular point of 14.7K action, especially as recruitment of 14.7K to TNFR1 could not be demonstrated. We therefore suggested an indirect mechanism of interference, probably involving cellular 14.7K interacting proteins. However, when analyzing caspase-8 activation in lysates from H1299 cells expressing 14.7K (Figure 2B and 6B), we found severely impaired processing of the full length form (p55/53) to the first intermediate product (p43/41) after TNF stimulation. Interestingly, this pointed to a mechanism acting directly at TNFR1 complex as initial cleavage of caspase-8 cleavage occurs receptor-associated [4], [6], [21], [22].

As our previous analysis of magnetically labeled TNRF1-containing endosomes did unfortunately not allow identification of 14.7K at TNFR1 level, we opted for an alternative experimental approach and performed immunoprecipitation experiments. Technically, detection of 14.7K expression in previous experiments was handicapped by restricted availability and limited shelf life of 14.7K antibodies used in studies over 20 years ago. This was further aggravated by the lack of commercially available, highly-sensitive antibodies. To overcome these issues, we used a custom-made polyclonal antibody from GST-14.7K immunized rabbits.

In immunoprecipitation experiments, we were for the first time able to demonstrate a ligand-inducible association of 14.7K, optineurin and TNFR1 (Figure 1). Interestingly, interaction of 14.7K and optineurin occurred even in the absence of TNF. This may reflect an artifact caused by supraphysiological expression levels of optineurin, but nevertheless provides evidence for a direct interaction of both molecules in a cellular system. These findings extended results from our previous study, which suggested an indirect interference of 14.7K involving cellular binding proteins. A yeast-two-hybrid screen identified optineurin as a 14.7K interacting protein [9], and this molecule was also found in a complex of TNFR1 and ubiquitinated RIP1 [11]. It was therefore tempting to speculate that optineurin may act as an adaptor molecule to convey 14.7K to activated TNFR1 complex, especially as our data from the mammalian-two-hybrid assay (Figure 3A) and coimmunoprecipitations (Figure 3B) confirmed predictions from earlier studies [9], [11].

Upon treatment with TNF, we observed formation of a protein complex containing TNFR1, ubiquitinated RIP1, optineurin and 14.7K. Integration of optineurin into the TNFR1 signaling complex has been demonstrated before [11], suggesting that ubiquitinated RIP1 acts as an adaptor protein for optineurin. Detection of RIP1 in its ubiquitinated form is indicative for ongoing early events in NF-κB activation.

Since association of optineurin with the TNFR1 complex was unaffected by the presence of 14.7K, the latter apparently does not interfere with optineurin recruitment and RIP1-optineurin binding (Figure 1A). Of note, binding sites for ubiquitinated RIP1 and 14.7K were both mapped to the C-terminus of optineurin [8] but seemingly did not interfere with each other as otherwise TNFR1 recruitment of one of the components is likely to be impaired. Still, it was possible that the pool of immunoprecipitated optineurin contained optineurin-RIP1-TNFR1 complexes as well as cytosolic optineurin-14.7K complexes. To circumvent this, we precipitated biotinylated TNF bound to TNFR1, thereby excluding non-TNFR1-associated cytosolic protein complexes (Figure 1B). A distinct recruitment of 14.7K to TNFR1 was found in a TNF-dependent manner and interaction of 14.7K with TNFR1 complex was again confirmed. Interestingly, even in the absence of TNF and therefore missing ligand-induced optineurin recruitment to TNFR1, a small amount of TNFR1 complex associated 14.7K was detectable (Figure 1B). This finding was verified in another immunoprecipitation experiment (Figure 7C), in which after siRNA-mediated knockdown of optineurin TNFR1-14.7K interaction was still observed. Together, these data indicated that optineurin-14.7K interaction is not a canonical prerequisite for recruitment of 14.7K to TNFR1.

To further investigate the role of 14.7K and optineurin recruitment to TNFR1 after ligand binding, we took in a first approach advantage of a previously established point mutation of 14.7K [14]. Loss of protein function due to a C119S substitution resulted in apoptotic cell death after TNF exposure (Figure 2), with activation of initiator and effector caspases, a hallmark of apoptosis. TNF-sensitivity of 14.7K PM expressing cells is in line with previous studies [4], [14], and has been attributed to a mutation related loss of function.

Interestingly, we recognized absent optineurin binding in TNF-sensitive 14.7K PM cell lines (Figure 3A–B), supporting our hypothesis for a crucial role of optineurin-14.7K complex in mediating TNF-resistance.

Unfortunately, 14.7K PM has several experimental limitations. The protein is reported to be unstable and therefore in Western blot analysis barely detectable, as substitution of the cysteine residue 119 disrupts sulfide bonds and affects zinc binding properties [19]. This tremendously complicates assessment of protein expression, and distinguishing between low protein levels and loss protein functions as correlate for the sensitivity towards TNF is difficult. Additionally, 14.7K PM does not allow discrimination between mutation-related loss of function or loss of optineurin binding as cause for TNF-sensitivity. As deletion of amino acids almost inevitably resulted in loss of protein function [14], our experimental approach based on systematic replacement of five amino acids with a Flag-epitope (DYKDE) (Figure 4). Analyzing optineurin binding of the generated 14.7K mutants revealed that amino acid substitutions in the C-terminus were accompanied with loss of binding capacity (Figure 5). This is in agreement with earlier data stating a crucial role of the C-terminus for structural integrity of 14.7K [14], [19]. The only exception seemed to be “14.7K mut 21”, with apparently conserved optineurin interaction in the mammalian-two-hybrid system. However, we were repeatedly not able to confirm the predicted interaction in immunoprecipitation experiments (data not shown), therefore an assay-derived artifact cannot be ruled out.

Pure physical interaction of 14.7K and optineurin was not sufficient to protect against TNF, as in cytotoxicity assays H1299/14.7K mut 3 cells died upon TNF-treatment in a dose-dependent manner (Figure 6A). Cell death involved cleavage of caspase-8 and caspase-3, thereby triggering the apoptotic pathway (Figure 6B). Finally, a substantial role for optineurin in 14.7K-mediated TNF-protection was ruled out in siRNA experiments. Even at not detectable optineurin levels in Western blot, 14.7K expressing cells were resistant to TNF (Figure 7B) and still exhibited blocked TNFR1 internalization (Figure 8).

Beside induction of cell death, TNFR1 signaling is under physiological circumstances mainly proinflammatory, especially through activation of the NF-κB pathway. Therefore, after excluding optineurin as a key player in 14.7K-mediated TNF-resistance and as previous studies provided evidence for a role of optineurin in NF-κB signaling [11], [17], [23], we investigated the effects of optineurin-14.7K interaction in this pathway. Interestingly, Sudhakar and colleagues characterized optineurin itself as a target gene of NF-κB [23], [24]. Optineurin recruitment to TNFR1 has been postulated to negatively regulate TNF-induced NF-κB activation due to competition of optineurin with the regulatory subunit of IκB kinase (NF-κB essential modulator, NEMO) for ubiquitinated RIP1 [11]. Nagabhushana and colleagues proposed a more detailed mechanism with optineurin as adaptor molecule for deubiquitinases like CYLD to terminate NF-κB signaling by inactivation of RIP1 [17]. Though, optineurin might be part of a negative feed back loop for proinflammatory TNFR1 signaling. Consequently, downregulation of endogenous optineurin should cause higher levels of phospho-p65 and IL-8 due to prolonged NF-κB signaling. However, in our experiments knockdown of optineurin did not result in sustained NF-κB signaling as measured by comparable amounts of IL-8 production and p65 phosphorylation in optineurin-naïve or knockdown cells (Figure 9B–C). In addition, with respect to IκBα phosphorylation we could not detect enhanced NF-κB activation in optineurin knockdown cells (Figure 9A) as reported previously [23]. The fact that rapid IκBα phosphorylation as indicator for early steps in NF-κB activation occured in 14.7K protected cells with blocked TNFR1 internalization is consistent with earlier reports claiming activation of this proinflammatory pathway at plasma membrane level in the absence of receptor internalization [4], [6], [21].

Together, our results did not point towards a regulatory role of optineurin in TNFR1-induced NF-κB activation. This discrepancy may arise from cell line specific properties in NF-κB signaling and different experimental design. Sudhakar et al. analyzed NF-κB activation using a reporter gene assay [23], whereas we measured endogenous IL-8 production. The latter might be also regulated through complementary mechanisms or overlapping regulatory feedback loops.

In summary, ligand-inducible association of optineurin and 14.7K with TNFR1 complex is not linked to 14.7K-mediated TNF-resistance. Protection against cytotoxic effects exclusively depends on expression of functional 14.7K, whereas interaction with optineurin in this regard is dispensable. Concerning activation of the NF-κB pathway, we found that recruitment of 14.7K to TNFR1 does not impair TNF-induced NF-κB activation. However, further studies are needed to clarify the impact of optineurin-14.7K complex on TNFR1-induced NF-κB signaling, especially in context of viral infection.

Perspectively, understanding the precise molecular mechanism underlying 14.7K-mediated apoptosis blockade could be exploited as a therapeutic strategy for treatment of disorders associated with increased TNF-levels such as rheumatoid arthritis and Crohn’s disease. In this context, blocking deleterious TNF-effects could control cytokine-induced collateral damage in inflammed tissue.

## Materials and Methods

### Cell Culture, Plasmid and siRNA Transfections

H1299 cells were obtained from LGC Standards GmbH (Wesel, Germany), HEK293 and KB cells were purchased from the German collection of microorganisms and cell cultures (Leibnitz Institute, Braunschweig, Germany). H1299, HEK293 and KB cells were grown in Dulbecco’s modified Eagle’s medium (DMEM; Invitrogen, Carlsbad, USA) supplemented with 10% fetal bovine serum, 100 U/mL penicillin and 100 µg/mL streptomycin. Transient transfections of plasmids used polyethyleneimide (Polysciences, Warrington, USA). In brief, cells were seeded at 80% confluency overnight. Before transfection, growth medium was replaced with DMEM without fetal bovine serum and antibiotics. Polyethyleneimide stock solution (1 mg/mL) was mixed with plasmid DNA at a 3∶1 ratio in 1 mL DMEM and incubated for 20 min at room temperature before added to the cells. Cells were incubated for 4–6 hours before medium was replaced by growth medium supplemented with 10% fetal bovine serum, 100 U/mL penicillin and 100 µg/mL streptomycin.

Knockdown of optineurin was performed using specific siRNAs (Dharmacon, Lafayette, USA). Briefly, cells were seeded in 6-well plates (1.5×10^5^ per well) and transfected with 100 pmol of specific or control siRNA using Lipofectamine 2000 transfection system (Invitrogen, Carlsbad, USA) according to manufacturer’s instructions. Two days post transfection, knockdown efficacy was determined by Western blotting. Subsequent cytotoxicity assays were performed in 96-well plates.

### Retroviral Transfections of 14.7K cDNAs

H1299 and KB cells were transduced with pQCXIP (Clontech, Cambrex, USA) expressing 14.7K or 14.7K mutants and the packaging vector pCL10A1 (Imgenex Retromax System, Biomol, Hamburg, Germany), essentially as described elsewhere [6]. After 2–3 rounds of serial transductions, transduced cells were selected using puromycin (3 µg/mL).

### Antibodies

Rabbit polyclonal anti-optineurin (Abcam, Cambridge, UK), anti-HA-antibody (Dianova, Hamburg, Germany), anti-α-Tubulin (Santa Cruz, CA, USA), anti-RIP1 (BD Biosciences, Heidelberg, Germany), anti-TNFR1, anti-Caspase-8 and anti-Caspase-3 were obtained from Cell Signaling (Beverly, MA, USA). Anti-14.7K is a selfmade polyclonal antibody from GST-14.7K immunized rabbits (Immunoglobe, Himmelstadt, Germany). HRP-conjugated anti-mouse and anti-rabbit antibodies were purchased from Biorad (Munich, Germany).

### Expression Vectors and Plasmid Construction

14.7K mutants were generated using the PCR-based “QuickChange” site-directed mutagenesis kit (Stratagene, Santa Clara, USA), sequentially replacing five amino acids by a Flag-tag sequence (DYKDE). 14.7K PM was generated as described previously [4]. Expression of 14.7K constructs used the pQCXIP vector system (BD Biosciences).

For the mammalian-two-hybrid screen 14.7K constructs were cloned into pCMV-AD vector (Stratagene) in frame with a N-terminal p65-tag. Truncated OPTN Δ1-394 was generated by PCR (primers: forward 5′-GAA TTC ATG AAG CTT CTG CAA GAA CAT AAT AAT G-3′, reverse 5′-GTC GAC TTA CGT GCC AGT GGA GAC TGT TC-3′) and cloned into pCMV-BD vector with a N-terminal Gal4-tag. For immunoprecipitations, HA-tagged or untagged variants of 14.7K and optineurin were expressed using the pQCXIP vector system. Full length optineurin was kindly provided by Dr. Stephen Ferguson (Robarts Research Institute, London, UK).

### Mammalian-two-hybrid Assay

The mammalian-two-hybrid assay was purchased from Stratagene and performed according to manufacturer’s instructions. Briefly, 4×10^5^ HEK293 cells grown on 6-well plates were transfected with 200 ng of pCMV-AD and pCMV-BD containing OPTN Δ1-394 or a 14.7K construct. pFR-Luc was cotransfected (1200 ng) as reporter plasmid. Luciferase activity was measured with a commercial assay (Dual-Luciferase reporter Assay System, Promega, Mannheim, Germany) and normalized to Renilla activity from cotransfected pRL-TK (Promega).

### Coimmunoprecipitation

For coimmunoprecipitation experiments of optineurin and 14.7K, HEK293 cells (3×10^6^ cells per 10 cm dish) were transfected with 5 µg of OPTN-HA and 14.7K constructs. For coimmunoprecipitation experiments of HA-tagged 14.7K and 14.7K PM with optineurin, HEK293 cells were transfected with 5 µg 14.7K and 5 µg optineurin contructs. To achieve equal expression levels of HA-tagged 14.7K and 14.7K PM, 15 µg of 14.7K PM construct was transfected along with optineurin construct. Cells were harvested in lysis buffer containing protease inhibitor cocktail (complete EDTA free, Roche, Mannheim, Germany) and incubated 20 min on ice. Samples were cleared by centrifugation (2×20000×g, 15 min), and incubated with 40 µL of a 50% HA-agarose slurry (Sigma, Steinheim, Germany) at 4°C overnight shaking overhead. After washing in lysis buffer, agarose-bound proteins were elutated by incubation at 95°C in 2× Laemmli sample buffer (4% SDS, 10% 2-mercaptoethanol, 20% glycerol, 0.125 M Tris, pH8, 0.004% bromphenol blue).

Immunoprecipitation of 14.7K was performed by adding 1 µg of specific antibody to the lysate, followed by incubation at 4°C overnight shaking overhead. Protein G sepharose (GE Healthcare, Uppsala, Sweden) was washed three times in lysis buffer and 40 µL of a 50% slurry were added for another 1–3 h and shaked overhead. After washing with lysis buffer, sepharose-bound proteins were eluted by incubation at 95°C for 5 min in 2× Laemmli sample buffer.

### TNFR1 Internalization Assay

1×10^5^ H1299 cells were seeded on coverslips. Biotinylated TNF and strepatvidin-FITC reagent (R&D Systems, Wiesbaden, Germany) were mixed and incubated at roomtemperature for 60 min in the dark.

Cells were incubated with biotin-TNF/streptavidin-FITC-complex for 60 min on ice in the dark. Cells were washed with ice-cold PBS and placed into 37°C pre-warmed medium for another 60 min to allow receptor internalization. For analysis of TNF-receptor endocytosis, cells were fixed with paraformaldehyde (2% in PBS) for 15 min and prepared for immunfluorescence. For analysis, a confocal laser scanning microscope was used (LSM 510 META, Zeiss).

### Precipitation of TNFR1-complexes

For coimmunoprecipitation of TNFR1-complex proteins, KB cells were seeded at a density of 1×10^7^ cells in a 15 cm dish and transfected with 30 µg of OPTN-HA, using polyethyleneimide transfection method (see above). 24 hours after transfection, cells were stimulated with 50 ng/mL of unlabeled TNF (Knoll AG, Ludwigshafen, Germany) or biotin-labeled TNF (R&D Systems, Minneapolis, USA) for 10 min or left untreated. Cells were washed with ice-cold PBS and harvested in lysis buffer containing protease inhibitor cocktail (complete EDTA free, Roche, Mannheim, Germany). Cell lysates were incubated 20 min on ice and cleared by centrifugation (2× 20000×g, 15 min). In case of stimulation with unlabeled TNF, lysates were incubated with 40 µL of a 50% HA-agarose slurry (Sigma, Steinheim, Germany) at 4°C overnight. In case of stimulation with biotin-TNF, 40 µL of a 50% streptavidin-agarose slurry was added to the lysates. Lysates from unstimulated cells supplemented with 50 ng/mL TNF or biotin-TNF served as negative control. After washing in lysis buffer, agarose-bound proteins were eluted by incubation at 85°C for 15 min in 2× Laemmli sample buffer. In case of coimmunoprecipitation of TNFR1-complex proteins in siRNA-optineurin knockdown cells, KB cells were seeded at a density of 2×10^6^ in a 10 cm dish and transfected with 600 pmol optineurin-specific siRNA or control siRNA. 24 hours after transfection, cells were stimulated with 50 ng/mL biotin-labeled TNF for 10 min or left untreated. Following procedures were performed as indicated above.

### Western Blotting

Cells were washed with ice-cold PBS, harvested in lysis buffer supplemented with protease inhibitor cocktail and incubated 20 min on ice. Lysates were cleared by centrifugation (20000×g, 20 min) and protein concentration was determined using Bradford reagent (Biorad, Munich, Germany). Laemmli sample buffer was added and samples were boiled for 5 min at 95°C. Proteins were separated by sodiumdodecylsulphate-polyacrylamide gel electrophoresis (SDS-PAGE) and transferred to PVDF membranes (Roche, Diagnostics, Indianapolis, USA). After blocking non-specific binding sites by incubation in Tris-buffered saline containing 0.1% Tween-20 and 5% dry milk, primary antibodies of indicated specificity were added. Subsequent incubation with horseradish peroxidase-conjugated secondary antibodies (Biorad) allowed detection of protein bands using SuperSignal West Substrate (Pierce, Bonn, Germany).

### TNF Cytotoxicity Assays

Cells were seeded in a 96 well plate in triplicates (3×10^4^ cells/well), pre-treated with cycloheximide (H1299 cells 12.5 µg/mL, KB cells 2.5 µg/mL) and finally challenged with increasing amounts of TNF (H1299 cells 0.001–10 ng/mL, KB cells 0.001–1000 ng/mL) (Knoll AG, Ludwigshafen, Germany) overnight. In case of siRNA treatment cells were challenged with final concentration of 1 ng/mL TNF. Vital cells were stained with crystal violet (0.5% crystal violet, 4% formaldehyde, 30% ethanol, 30 mM NaCl) and solved in 200 µL 30% acetic acid. Optical density was analyzed at 595 nm in a photometer.

### ELISA

Cells were seeded in 6-well plates (1.5×10^5^ per well) and transfected with 100 pmol of specific or control siRNA using Lipofectamine 2000 transfection system (Invitrogen, Carlsbad, USA) according to manufacturer’s instructions. For IL-8 detection, cells were treated 24 hours after transfection with 20 ng/mL TNF for another 24 hours. Supernatants were harvested and subjected to IL-8 ELISA (BD Biosciences, Heidelberg, Germany) according to manufacturer’s instructions. For detection of phosphorylated NF-κB subunit p65, cells were treated 48 hours after transfection with 20 ng/mL TNF for 10 min, harvested and subjected to phospho-p65 ELISA (Cell Signaling, Beverly, MA, USA) according to manufacturer’s instructions.
